# Neural Dynamics of Improved Bimodal Attention and Working Memory in Musically Trained Children

**DOI:** 10.3389/fnins.2020.554731

**Published:** 2020-10-08

**Authors:** Leonie Kausel, Francisco Zamorano, Pablo Billeke, Mary E. Sutherland, Josefina Larrain-Valenzuela, Ximena Stecher, Gottfried Schlaug, Francisco Aboitiz

**Affiliations:** ^1^Centro Interdisciplinario de Neurociencias, Pontificia Universidad Católica de Chile, Santiago, Chile; ^2^Laboratorio de Neurociencia Social y Neuromodulación, Centro de Investigación en Complejidad Social (CICS), Universidad del Desarrollo, Santiago, Chile; ^3^Unidad de Imágenes Cuantitativas Avanzadas, Cl nica Alemana, Universidad del Desarrollo, Santiago, Chile; ^4^Departamento de Imágenes, Cl nica Alemana, Universidad del Desarrollo, Santiago, Chile; ^5^Neuroradiology, Radiology Department, Clinica Alemana de Santiago, Santiago, Chile; ^6^Department of Neurology, Beth Israel Deaconess Medical Center and Harvard Medical School, Boston, MA, United States

**Keywords:** attention, working memory, fronto-parietal control network, phonological loop, musical training

## Abstract

Attention and working memory (WM) are core components of executive functions, and they can be enhanced by training. One activity that has shown to improve executive functions is musical training, but the brain networks underlying these improvements are not well known. We aimed to identify, using functional MRI (fMRI), these networks in children who regularly learn and play a musical instrument. Girls and boys aged 10–13 with and without musical training completed an attention and WM task while their brain activity was measured with fMRI. Participants were presented with a pair of bimodal stimuli (auditory and visual) and were asked to pay attention only to the auditory, only to the visual, or to both at the same time. The stimuli were afterward tested with a memory task in order to confirm attention allocation. Both groups had higher accuracy on items that they were instructed to attend, but musicians had an overall better performance on both memory tasks across attention conditions. In line with this, musicians showed higher activation than controls in cognitive control regions such as the fronto-parietal control network during all encoding phases. In addition, facilitated encoding of auditory stimuli in musicians was positively correlated with years of training and higher activity in the left inferior frontal gyrus and the left supramarginal gyrus, structures that support the phonological loop. Taken together, our results elucidate the neural dynamics that underlie improved bimodal attention and WM of musically trained children and contribute new knowledge to this model of brain plasticity.

## Introduction

Executive functions, which include goal-directed attention and working-memory capacity, allow us to regulate, control, and manage our thoughts, emotions, and decision making (Aboitiz and Cosmelli, 2009). Attention allows us to select the stimuli that are relevant for us at each moment, and working memory (WM) allows us to keep the information in an accessible state for a short time. Attention and WM are closely related, because paying attention to certain information makes it easier to remember (Chun and Turk-Browne, 2007; Fougnie, 2008). Switching attention from one task to another also requires cognitive flexibility, an ability that helps to adjust one’s behavior according to a changing environment (Monsell, 2003; Armbruster et al., 2012), and which is a core component of executive functions (Diamond, 2013). Greater cognitive flexibility is associated with favorable outcomes throughout lifespan, such as higher resilience, improved reading abilities in childhood, higher creativity, and a better quality of life (Dajani and Uddin, 2015). These skills, used every day to interact with our world (Hinton et al., 2012), develop during childhood and adolescence and can be improved by training (Diamond, 2013).

One activity that has been proposed to improve executive functions is playing a musical instrument (Miendlarzewska and Trost, 2014). Playing a musical instrument is a very challenging activity that puts high demands on motor and multisensory skills and is usually begun at an early age. Musicians have to master independent motor control for each hand, listen to what they play, react to what they hear, and also pay attention to other players (when playing in an ensemble). Score reading, which implies transforming visual symbols into auditory patterns by means of playing the instrument, is also part of most musical trainings. It has been shown that musical training produces structural and functional changes in the brain. As such, it has been proposed as a model for the study of brain plasticity (Schlaug, 2015).

Research has shown that adult musicians outperform their untrained peers on tasks assessing cognitive flexibility, WM, and verbal fluency (Zuk et al., 2014). Increased WM capacity has also been found in musically trained children and adolescents, with improved visuo-spatial and verbal WM and improved processing speed and reasoning (Bergman Nutley et al., 2014). In this latter study, researchers also found that changes in WM were proportional to the weekly hours spent on music practice. Still, results that show that better performance of musically trained children on visual WM tasks has not always been consistent (Talamini et al., 2017). Ho et al. (2003) found that children with musical training had better verbal memory, but not visual memory. Importantly, the improvements in verbal memory were maintained in those students who had begun or continued musical training after a year. Also, there is some behavioral evidence from young adults that musical training enhances task switching and dual task performance (e.g., Moradzadeh et al., 2015), which are tasks that require high performance of executive functions. Furthermore, it has been shown that musically trained young adults have higher efficiency of the executive attention network, which is involved in top-down attentional control (Medina and Barraza, 2019). It has also been shown that musical training has a positive impact on children who have auditory- and attention-related developmental disorders [attention deficit hyperactivity disorder (ADHD) or dyslexia], improving their neural efficiency of auditory cortex and promoting intrahemispheric synchronization (Seither-Preisler et al., 2014; Serrallach et al., 2016). Even though it has been shown that musically trained children have enhanced executive functions, the neural dynamics underlying these improvements are not well known.

Recent brain imaging studies have shown that musically trained children have higher activation of the bilateral supplementary motor area (SMA), the inferior frontal gyrus (IFG), the anterior cingulate cortex and the insula in a visual Stroop task (Sachs et al., 2017), and the pre-SMA/SMA and the right ventrolateral prefrontal cortex in a set-shifting task (Zuk et al., 2014). A study in young adult musicians showed that they had higher activation than a control group in cognitive control-related areas such as the bilateral posterior dorsal prefrontal cortex and the anterior cingulate gyrus when solving a musical-sound WM task (Pallesen et al., 2010). These studies suggest that it is plausible that musical training could influence the neural networks that underlie better performance of executive functions in musically trained children.

In order to better determine the neural dynamics involved in performance of musically trained children in executive function tasks, particularly in a bimodal context, our study sought to determine the neural correlates that underlie bimodal auditory/visual attention and WM in musically trained children. We hypothesized that playing a musical instrument improves these functions and that the neural networks underlying these skills would be boosted in children who regularly learn and play a musical instrument.

In the present study, we used functional magnetic resonance imaging (fMRI) to investigate the influence of musical training on the neural correlates that underlie bimodal attention and WM in musically trained children. To achieve our goal, we adapted and implemented the bimodal attention task of Johnson and Zatorre (2006). Participants were presented with a simultaneous pair of bimodal stimuli (auditory and visual) and were asked to pay attention only to the auditory, only to the visual (selective attention), or to both at the same time (divided attention). Both stimuli were afterward tested with a memory task in order to confirm attention allocation. By combining behavioral measures and brain activity recordings, we were able to determine the neural dynamics underlying the improved performance of musically trained children on our task.

## Participants and Methods

### Participants

Forty healthy, right-handed, Spanish-speaking children aged 10–13, with normal hearing and normal or corrected-to-normal vision, participated in our study. Written informed consent was obtained from all children and their parents for a protocol approved by the ethics committee of the Pontificia Universidad Católica de Chile. Participants completed the Wechsler Intellectual Scale for Children (WISC III) (Wechsler, 1991) validated for Chilean population (Ramírez and Rosas, 2007) and answered the Spanish version of the standardized Montreal Music History Questionnaire (Coffey et al., 2011), which inquired about their personal experience in music listening and performing. In a second session, participants solved the bimodal selective and divided attention task while their brain activity was being measured with fMRI. Participants received monetary compensation for travel costs.

Twenty musically trained participants were recruited from different youth orchestras in Santiago, Chile. Inclusion criteria encompassed playing a melodic instrument, having at least 2 years of instrumental lessons, practicing at least 2 h/week, and regularly playing in an orchestra or an ensemble. Six children played wind instruments (three clarinets, one traverse flute, one horn, and one saxophone), and 14 played string instruments (12 violins, one viola, and one cello). Age of onset of musical training was 9.1 ± 1.6 years (range from 6 to 11), average musical training was 3.7 ± 1.3 years (range from 2 to 6 years), intensity of practice over the last year was 9.2 ± 5.3 h/week (range from 2 to 21), and all participants had studied music continuously since the onset of training. All children were trained based on more non-aural strategies and had individual or small group (two to three participants) instrumental lessons and also played in an orchestra, having rehearsals at least once a week over the last year. Twenty control children were recruited from public schools in Santiago and had no additional musical training than the one provided in school curricula. In contrast to musically trained children, control children all declared to be unable to read or write musical scores.

Importantly, groups were matched for gender, age, intelligence coefficient (WISCIII), and socioeconomic status (educational level of both parents) (Table 1). For parental education, the highest, successfully completed education level of the parents was re-coded into a measure reflecting level of education, ranging from 1 (incomplete middle school education) to 10 (complete PhD). The average of both parents was used (Liberatos et al., 1988). The guardian of one musically trained child did not provide father’s education, and the guardian of one control child did not provide parental education.

**TABLE 1:** General demographics of the study population.

	**Musically trained children**	**Control children**	
**n**	18	17	
Females	10	11	

	**Mean ± *SD***	**Mean ± *SD***	***t-*value (*p*-value)**

Age (years)	12.2 ± 0.8	12.2 ± 0.8	−0.08(0.53)
IQ	109.5 ± 10.3	105.9 ± 11.1	0.96 (0.17)
Parental education	3.8 ± 1.7	4.1 ± 1.6	−0.65(0.74)

**There were no significant differences between groups for age, IQ, and parental education. IQ, intelligence coefficient.**

Five participants were excluded because of excessive movement during scanning. Finally, 18 musically trained children (10 female, mean age = 12.2 ± 0.8 years) and 17 non-musically trained children (11 female, mean age = 12.2 ± 0.8 years) were included in the analysis (Table 1). Table 2 shows the musical training details of the musically trained children who were included in the analysis.

**TABLE 2:** Characteristics of musical training in musically trained children.

**Musically trained children (*n* = 18)**		
**Group characteristics**	**Mean ± *SD***	**Range**
Age at onset of musical training (years)	9.1 ± 1.6	6–11
Intensity of practice over the last year (hours/week)	9.2 ± 5.3	2–21
Duration of musical training (years)	3.7 ± 1.3	2–6

**Type of musical instrument**	**Number of children**	

Strings	13	
Woodwinds	3	
Brass	2	

### Experimental Paradigm and Stimuli

#### Experimental Task

The bimodal (auditory/visual) attention task that was used was adapted from Johnson and Zatorre (2006). In particular, we adapted the length of the stimuli by making them shorter (4 s) and adding the memory retrieval task after each stimulus pair. Participants solved this task while their brain activity was measured with fMRI.

#### Stimuli

Auditory (melodies) and visual (figures) stimuli were 4 s long. We included a defined feature to the stimuli—a chord in the melody and a line of a different color in the figure—in order to help children to direct their attention to only one modality during the selective attention conditions. They were asked to report the chord by button press during the encoding phase of the auditory selective attention condition (ASA) and the red line during the visual selective attention condition (VSA). Melodies were in major tonalities and comprised pitches drawn from the Western musical scale centered around the mid-range of the piano from F3 (175 Hz) to G6 (784 Hz), with quarter and eighth notes. They were all in wav format and were presented in a piano timbre. All melodies contained one chord, which had to be reported by button press during the ASA. The melodies were presented binaurally at a comfortable listening level for each subject through MR-compatible sound transmission headphones (Resonance Technology Inc.)^1^. Figures consisted of equally long nine black lines and one red line, which had to be reported by button press during VSA. In order to “draw” each figure on a white background, individual shapes had the same starting point and new lines were presented sequentially aligned either horizontally or vertically every 300 ms. An abstract shape formed by 10 consecutively incorporated lines was completed after 3,000 ms and remained in view for 1,000 ms. The MRI head coil had a mirror attached, so that participants could see the screen where visual stimuli were displayed. A total of 160 melodies and figures were created. When presented simultaneously the auditory and visual stimuli, started and stopped at exactly the same time, but the individual elements of the two stimuli never synchronized. Stimuli were presented using Presentation Software (Neurobehavioral Systems).

#### Procedure

**FIGURE 1:**
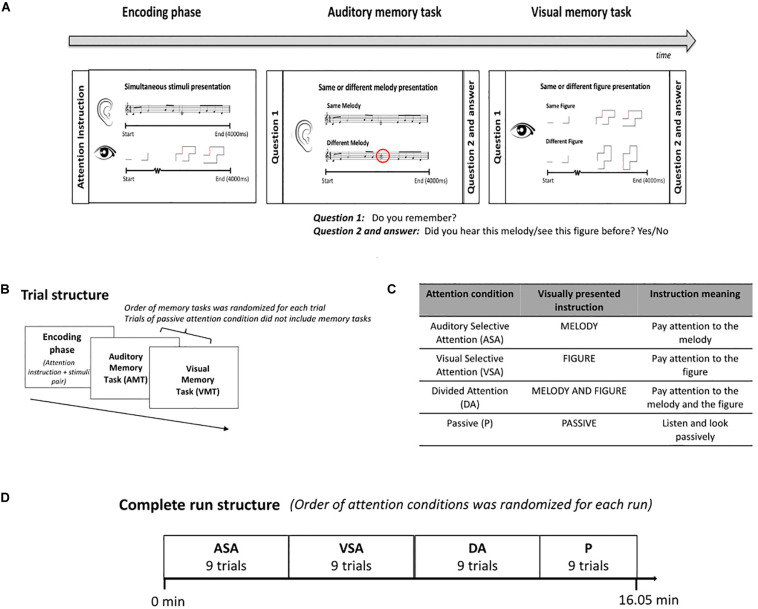
Experimental protocol. **(A)** Scheme of the structure of the task and examples of the stimuli presented during the encoding phase and the retrieval memory tasks. **(B)** Trial structure. ASA, VSA, and DA included encoding phase and retrieval memory tasks. Order of memory tasks was randomized for each trial. P only included encoding phase. **(C)** The attention condition of each trial was given by the instruction presented at the beginning of the encoding phase. **(D)** Complete run structure. Order of attention conditions was randomized for each run. All participants completed one run of the task in the MRI scanner. ASA, auditory selective attention condition; VSA, visual selective attention condition; DA, divided attention condition; P, passive condition; VMT, visual memory task; AMT, auditory memory task.

Each trial of our task had two parts, the encoding phase and the memory retrieval tasks (Figure 1A). The encoding phase started with an instruction to pay attention to either (or both) the melody or figure and then presented a pair of stimuli, which included an evolving abstract figure (visual) and a melody (auditory). Both stimuli lasted 4 s and started and stopped at exactly the same time, but the individual elements of the two stimuli never synchronized. Where attention was directed to was given by attention instruction (Figure 1C) and defined the auditory selective (ASA), visual selective (VSA), and divided attention (DA) conditions, respectively. We also included one condition in which children were instructed to passively observe the stimuli. This was the passive condition (P), and these trials did not include the memory retrieval tasks. The same/different memory retrieval tasks for both the auditory and visual stimuli followed each active attention encoding phase with a delay of 1,600 ms. These tasks allowed us to evaluate attention allocation (Figure 1B). Participants had 2.5 s to respond and had to report their answer via button press. Children did not receive any specific instruction on how to press the button (e.g., “respond as fast as possible”). During training outside of the scanner, we explained to the children that since we were interested in studying attention allocation, the most important thing during the experiment was that they followed the attention instruction of each attention condition. Better performance on the memory task for the attended stimuli was expected. Accuracy (correct responses) and reaction time of correct responses on the retrieval memory tasks were our behavioral outcome measures.

The trials in the same attention condition were presented as a block (Figure 1D). The order of attention condition blocks was randomized across participants. All conditions included unique stimuli, and stimulus pairs presented during encoding phases were defined randomly for each subject. The task had a duration of 16.05 min and included 36 trials, nine trials for each attention condition. All participants completed the task while being scanned in the MRI machine.

### Data Acquisition

Images were acquired at the Radiology Department of the Clínica Alemana de Santiago with a 3T Siemens Skyra scanner and a 20-channel head coil. Participants were prepared for the MRI and were instructed to relax and keep still during image acquisition. For each subject, a 3D structural T1-weighted scan [voxel size, 1 × 1 × 1 mm; slices per slab, 176; field of view (FoV), 256 mm; repetition time (TR) = 2.53 s; echo time (TE) = 2.19 ms], phase and magnitude field maps (voxel size, 2.7 × 2.7 × 2.3 mm; slices, 72; FoV, 208; TR = 731 ms; TE1 = 4.92 ms; TE2 = 7.38 ms) and a functional T2^∗^-weighted gradient echo planar imaging scan (voxel size, 3 × 3 × 3 mm; slices, 38; FoV, 220; TR = 2.21 s; TE = 30 ms) were acquired.

During functional T2^∗^-weighted gradient echo planar imaging, our bimodal attention task was presented using Presentation Software (Neurobehavioral Systems). Auditory stimuli were presented over MRI-compatible headphones (Resonance Technology Inc.)^2^, and visual stimuli were presented on a screen located in the MRI room at the same viewing distance for all subjects. The coil had a mirror attached, so that participants could see the screen where visual stimuli were displayed. Answers were given via button press on a keypad.

### Data Analysis

#### Behavioral Data

Behavioral data were studied using RStudio (R Version 3.1.2). Accuracy and reaction time for memory tasks were analyzed with a 2 × 3 × 2 mixed analysis of variance (ANOVA) to compare the main effects and interactions of group (between-subject factor: musicians, controls), attention condition (within-subject factor: ASA, VSA, DA), and retrieval memory task [within-subject factor: visual memory task (VMT), auditory memory task (AMT)]. Whenever the assumption of sphericity was violated, the Greenhouse–Geisser correction for epsilon was applied. Interaction effects were further assessed with pairwise *t*-tests. Bonferroni correction for multiple comparisons was applied where necessary. Alpha level of 0.05 was used for all statistical tests.

#### Functional Magnetic Resonance Imaging

fMRI data were analyzed using FMRIB Software Library (FSL, version 5.0.10)^3^ (Smith et al., 2004; Jenkinson et al., 2012). Data preprocessing involved the following steps: motion correction including field map unwarping (MCFLIRT), slice timing corrections, brain extraction (BET), spatial smoothing with a 6 mm full width at half maximum (FWHM) Gaussian kernel, and high-pass temporal filtering using Gaussian-weighted least-squares straight line fitting with sigma = 100.0 s, and pre-whitening. The blood oxygenation level-dependent (BOLD) response was modeled using a separate explanatory variable (EV) for the encoding phase of each attention condition (ASA, VSA, DA, and P). The design was convolved with a double gamma hemodynamic response function and temporal derivatives of each EV time course, and motion correction parameters were included as additional nuisance regressors. Estimated beta maps for contrasts were normalized to MNI152 standard space using linear transformations (FLIRT) in two stages. First, functional images were aligned with the subjects’ high-resolution T1 using boundary-based registration (BBR). Then the T1 was registered to the standard Montreal Neurological Institute (MNI) atlas with a 12-degree-of-freedom affine transformation. Finally, these transformations were then applied to the functional data. Second-level activation maps were calculated with FSL using mixed-effect model (FLAME1 + 2). All reported results are based on an initial uncorrected voxel-level threshold of z > 3.1 and cluster inference using a familywise error-corrected threshold of *p* < 0.05, according to new MRI analysis guidelines (Eklund et al., 2016; Nichols et al., 2017).

In the first-level analysis, we modeled the encoding phases for each subject. The Hillyard principle (Hillyard et al., 1973) states that in order to assess the effects of directed attention, responses should be compared with the same physical stimuli while holding overall arousal level and task demands constant, such that all that differs is the focus of directed attention. See Figure 1C for instructions given in each condition. In short, children were instructed to pay attention to the figure (VSA), the melody (ASA), both the figure and the melody (DA) or to listen and look passively at the presented stimuli (P). We modeled the attention component of the encoding phases of the active attention conditions by subtracting the passive condition from the encoding phase of the other three attention conditions, resulting in the contrasts [ASA > P], [VSA > P], and [DA > P]. Note that the arousal level and task demand could be different between the active and passive conditions, because the passive condition was not followed by memory tasks. Nevertheless, this design was chosen considering that there is a tradeoff between arousal level and focusing attention, following previous literature (Johnson and Zatorre, 2006).

Then we carried out three second-level-analysis models. In the first one, we explored for differences between groups in encoding phases. In the second one, we added a regressor of the accuracy in the AMT of VSA trials to the [VSA > P] contrast, in order to further investigate the three-way interaction effect that we found in the behavioral analysis. In this model, we also explored for differences between groups. In a third model, we added a regressor for the time of musical training (years) to the [ASA > P] contrast in the musician group, in order to disentangle if the results obtained with the anterior model would be explained by a facilitation in the encoding of the auditory stimulus. Finally, we performed a conjunction analysis to determine the overlaps between (1) the contrasts that showed differences between musicians and controls in the encoding phases determined with the first model and (2) the results determined with the second and third models.

Activation maps selected for figures were overlaid on a high-resolution brain image in MRIcroGL or FSLeyes for visualization. Activation locations were confirmed using the Harvard-Oxford Cortical Structure Atlas. Data are presented following the radiological convention (L, left; R, right), and coordinates are in MNI space.

## Results

### Behavioral Results

**FIGURE 2:**
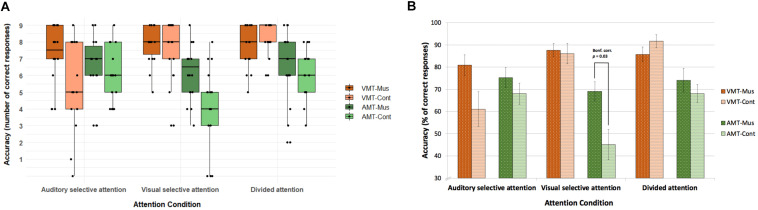
Accuracy on the retrieval memory tasks in the three active attention conditions. **(A)** Accuracy with individual data points for number of correct responses. **(B)** Accuracy in percentage of correct responses. Overall musically trained children had more correct responses than control children [*F*(1, 33) = 6.1, *p* = 0.019). The three-way interaction effect between group, attention condition, and memory task [*F*(3.1, 102.3) = 3.8, *p*[*GG*] = 0.047) was given by the correct responses to the AMT in the visual selective attention condition trials (Bonf. corr. *p* = 0.03). Error bars in **(B)** indicate standard error of the mean. VMT, visual memory task; AMT, auditory memory task; Mus, musically trained children; Cont, control children.

Accuracy and reaction times of correct responses for both groups for each memory task across attention conditions are shown in Figure 2 and Table 3, respectively. Results were analyzed with a 2 × 3 × 2 mixed ANOVA with group, attention condition, and retrieval memory task as factors.

**TABLE 3:** Reaction times for correct responses of the retrieval memory tasks after each attention condition.

**Attention condition before MT**	**VMT**		**AMT**	
	**Musicians**	**Controls**	**Musicians**	**Controls**
	**RT ± *SEM* (ms)**	**RT ± *SEM* (ms)**	**RT ± *SEM* (ms)**	**RT ± *SEM* (ms)**
ASA	808.6 ± 55.9	830.2 ± 118.3	835.8 ± 74.1	856.6 ± 74.0
VSA	772.1 ± 49.5	839.9 ± 54.3	788.3 ± 66.8	750.5 ± 123.8
DA	810.8 ± 52.7	777.1 ± 61.4	755.7 ± 47.4	765.4 ± 67.7

**There were no significant differences between reaction times. ASA, auditory selective attention; VSA, visual selective attention; DA, divided attention; VMT, visual memory task; AMT, auditory memory task.**

Our behavioral results showed an interaction effect among attention condition and memory task [*F*(3.1, 102.3) = 11.3, *p*[*GG*] = 0.0007, η^2^ = 0.09], which indicated that in both groups, attention condition significantly modulated the correct responses for memory tasks, with attended stimuli being better remembered than unattended ones. In other words, both groups remembered melodies better in auditory selective and divided attention conditions, whereas both groups remembered figures better in visual selective and divided attention conditions. This same modulation was found for adults in Johnson and Zatorre (2006), from where we adapted our task. Our results for correct responses to memory tasks also showed a significant main effect of group [*F*(1, 33) = 6.1, *p* = 0.019, η^2^ = 0.05]. Overall, musically trained children had a better performance on memory tasks than control children independent of attention condition (Mus: mean = 7.09, *SD* = 1.69; Cont: mean = 6.28, *SD* = 2.3). Finally, the three-way interaction that we found between group, attention condition, and memory task [*F*(3.1, 102.3) = 3.8, *p*[*GG*] = 0.047, η^2^ = 0.03] was given by the correct responses to the AMT in the VSA (*t* = 3.0226, *df* = 28.005, uncorrected *p* = 0.00531; Bonferroni-corrected *p* = 0.032). On average, musicians had 6.2 (*SD* = 1.6) correct responses as opposed to controls, who had an average of 4.1 (*SD* = 2.4) correct responses (Figure 2).

Behavioral results for reaction time of correct responses showed no significant main or interaction effects. There was no main effect of group [*F*(1, 33) = 0.02, *p* = 0.88] or interaction effects between group, attention condition, and memory task [*F*(2, 66) = 0.3, *p* = 0.74]. Overall mean reaction time was 809 ms (*SD* = 284 ms) (Table 3).

### Functional Magnetic Resonance Results

In order to determine the neural activity underlying attention during the encoding phases, we modeled our contrasts by subtracting the passive condition from the other three attention conditions. We expected that the main effect would be given by differences during the encoding phase, due to the role of attention on selecting the items that will be encoded in memory. The memory tasks were used to test the encoding process. We also explored if there were any differences during memory tasks, but we did not find any at our threshold levels (corrected *p* < 0.05).

**FIGURE 3:**
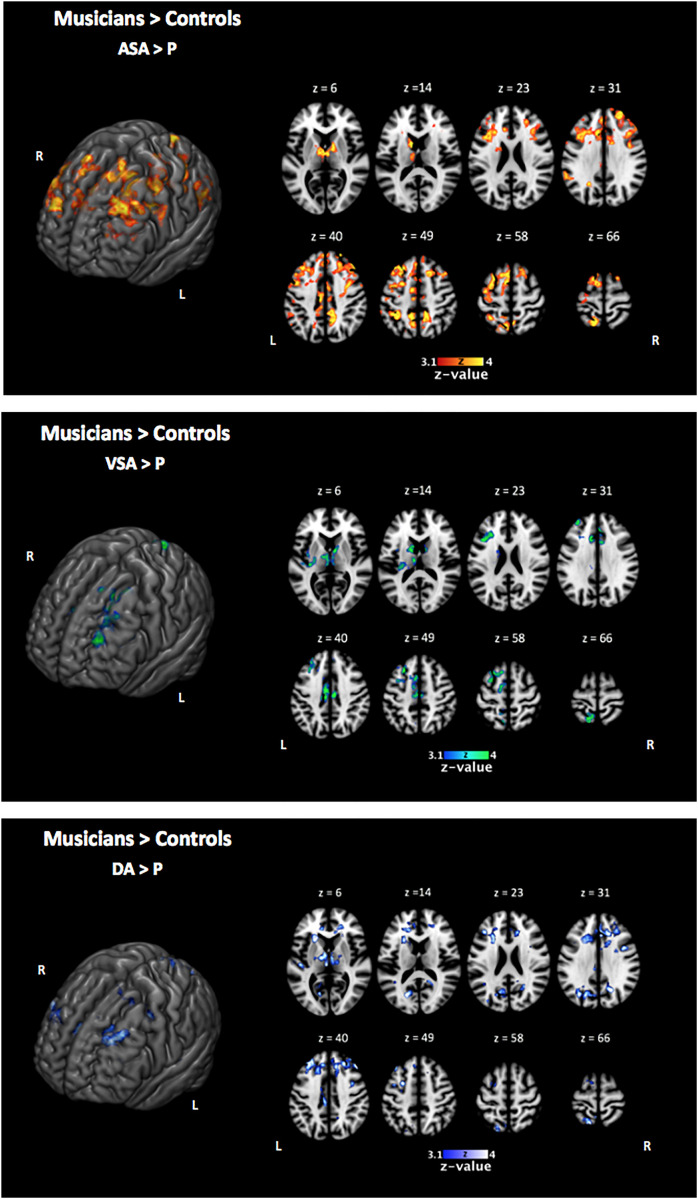
Two-sample comparison of musically trained children over control children during encoding phase [ASA > P], [VSA > P], and [DA > P] contrasts (corrected *p* < 0.05). ASA, auditory selective attention condition; VSA, visual selective attention condition; DA, divided attention condition; P, passive condition.

**FIGURE 4:**
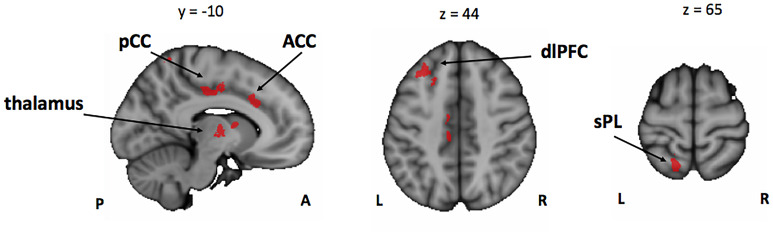
Conjunction analysis between the Mus > Cont contrasts of the encoding phase [ASA > P], [VSA > P], and [DA > P] contrasts. Mus, musically trained children; Cont, control children; ASA, auditory selective attention; VSA, visual selective attention; DA, divided attention; P, passive; pCC, posterior cingulate cortex; ACC, anterior cingulate cortex; dlPFC, dorsolateral prefrontal cortex; sPL, superior parietal lobe.

In line with our behavioral results that showed an overall better performance of musically trained children across attention conditions and memory tasks, whole-brain analyses of encoding phase contrasts ([ASA > P], [VSA > P], and [DA > P]) showed a significantly greater activation for musically trained children as compared with control children (corrected *p* < 0.05) in areas related to attentional control (Figure 3 and Table 4). Musically trained children showed higher activation in regions including bilateral dorsolateral prefrontal cortex (dlPFC), medial premotor area, right dorsal precentral gyrus (pre-CG), left supramarginal gyrus (SMG), bilateral posterior division of the cingulate cortex (PCC), and bilateral thalamus for the [ASA > P] contrast; left dlPFC, left superior parietal lobe (sPL), bilateral PCC and bilateral thalamus for the [VSA > P] contrast; and bilateral dlPFC, left sPL, left anterior division of the cingulate cortex (ACC), left PCC, and bilateral thalamus for the [DA > P] contrast. The conjunction analysis for these three Musicians > Controls contrasts showed an overlap in the left dlPFC, the left sPL, the ACC, the PCC, and the thalamus (Figure 4). The opposite comparison of control children over musically trained children resulted in no activation at our threshold level (corrected *p* < 0.05).

**TABLE 4:** Peaks of activity of group differences (Mus > Cont children) for the encoding phase contrasts [ASA > P], [VSA > P], and [DA > P].

**Area**	**x**	**y**	**z**	**Z-score**	**Cluster size**	**Corrected *p*-value**
**Mus > Cont ASA > P**						
Right precuneus	12	−42	47	5.19	83,684	2.32E–39
Right cuneal cortex	12	−82	35	5.07	83,684	2.32E–39
Left precentral gyrus	−41	−7	55	4.47	83,684	2.32E–39
Right cingulate gyrus, anterior division	4	−0.4	37	4.04	83,684	2.32E–39
Left middle frontal gyrus/superior frontal gyrus	−26	24	48	3.9	83,684	2.32E–39
Left superior frontal gyrus	−2	17	58	3.76	83,684	2.32E–39
Right middle frontal gyrus/superior frontal gyrus	21	24	48	3.6	83,684	2.32E–39
Left superior parietal lobe	−34	−47	59	3.5	83,684	2.32E–39
Left cingulate gyrus, anterior division	−2	0.3	36	3.35	83,684	2.32E–39
Left caudate	−10	5	14	5.08	5,731	3.34E–06
Left thalamus	−3	−7	5	5	5,731	3.34E–06
Right thalamus	4	−11	4	4.28	5,731	3.34E–06
Left supramarginal gyrus	−58	−42	36	4.6	2,111	0.00579
**Mus > Cont VSA > P**						
Right cingulate gyrus, posterior division	6	−18	45	4.94	15,899	2.69E–13
Left cingulate gyrus, posterior division	−7	−20	45	4.85	15,899	2.69E–13
Left paracingulate gyrus	−3	15	50	4.82	15,899	2.69E–13
Left superior frontal gyrus	−24	19	54	4.03	15,899	2.69E–13
Left middle frontal gyrus	−29	34	42	5.58	15,899	2.69E–13
Left caudate	−7	3	14	4.79	8,859	7.63E–09
Left thalamus	−7	−11	2	4.02	8,859	7.63E–09
Right thalamus	7	−12	2	3.48	8,859	7.63E–09
Left posterior insular cortex/planum polare	−39	−19	−4	5.16	5,502	2.62E–06
Left posterior insular cortex	−37	−17	−1	4.81	5,502	2.62E–06
Left medial insular cortex	−36	−5	−1	4.38	5,502	2.62E–06
Left putamen/pallidum	−25	−14	4	4.35	5,502	2.62E–06
Left cingulate gyrus, anterior division	−8	21	29	4.61	2,650	0.0011
Left lateral occipital cortex, superior division	−12	−60	67	4.53	2,405	0.00201
Left superior parietal lobe	−14	−55	69	4.39	2,405	0.00201
**Mus > Cont DA > P**						
Left middle frontal gyrus	−29	35	41	4.79	28,912	6.89E–19
Left paracingulate gyrus	2	35	33	4.77	28,912	6.89E–19
Left putamen	−22	21	5	4.74	28,912	6.89E–19
Right middle frontal gyrus	41	32	38	3.67	28,912	6.89E–19
Left cingulate gyrus, anterior division	−8	20	31	3.45	28,912	6.89E–19
Cuneal cortex	0	−76	28	4.73	11,088	9.34E–10
Left precuneus	−13	−68	25	4.7	11,088	9.34E–10
Left planum polare	−42	−23	−2	4.53	7,241	2.98E–07
Left parahippocampal gyrus, posterior division	−31	−24	−22	4.46	7,241	2.98E–07
Left thalamus	−14	−11	2	4.42	7,241	2.98E–07
Left pallidum	−26	−18	−1	4.42	7,241	2.98E–07
Right thalamus	12	−19	7	3.61	7,241	2.98E–07
Left lateral occipital cortex, superior division	−14	−61	65	4.63	2,757	0.00139
Left medial postcentral gyrus	−7	−48	73	4.47	2,757	0.00139
Left superior parietal lobe	−13	−55	71	3.37	2,757	0.00139
Left middle frontal gyrus/superior frontal gyrus	−25	4	52	4.4	2,055	0.00724
Left cingulate gyrus, posterior division	−5	−29	36	4.05	1,612	0.0225
Brain stem	5	−29	−13	4.51	1,388	0.0413

**Mus, musically trained children; Cont, control children; ASA, auditory selective attention condition; VSA, visual selective attention condition; DA, divided attention condition; P, passive condition.**

Taken together, our results suggest that the overall better performance of the musically trained children in our bimodal attention task seems to be driven by higher activation of attention control related brain areas from the fronto-parietal control network (e.g., dlPFC, sPL, and ACC) during encoding phase in musically trained children as compared with controls in all active attention conditions (Figure 4).

**FIGURE 5:**
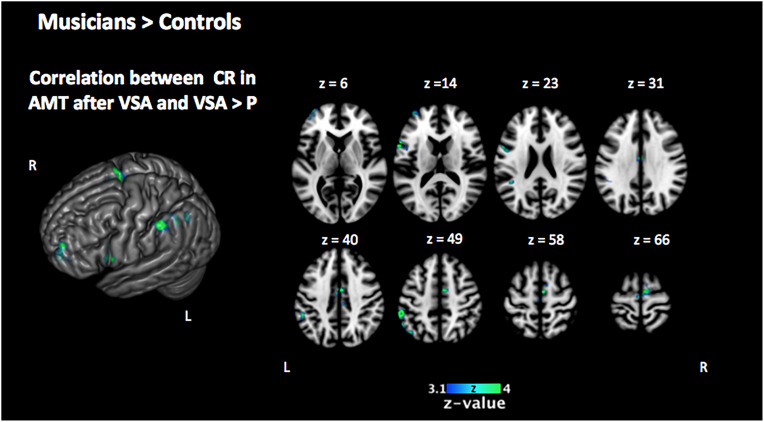
Two-sample comparison of Mus > Cont for the correlation between the activation in the encoding phase [VSA > P] contrast with the correct responses of each subject in the auditory memory tasks of the VSA condition trials (corrected *p* < 0.05). Mus, musically trained children; Cont, control children; VSA, visual selective attention; P, passive; CR, correct responses; AMT, auditory memory task.

**FIGURE 6:**
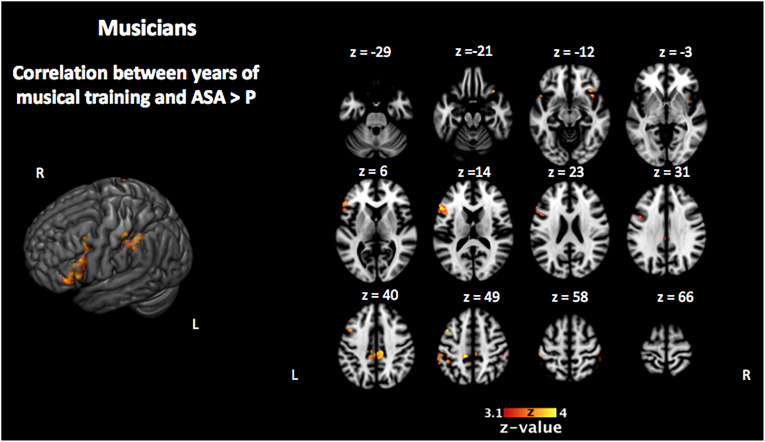
Activation that correlated with the years of musical training in the musically trained group during the encoding phase of the [ASA > P] contrast (corrected *p* < 0.05). ASA, auditory selective attention; P, passive.

**FIGURE 7:**
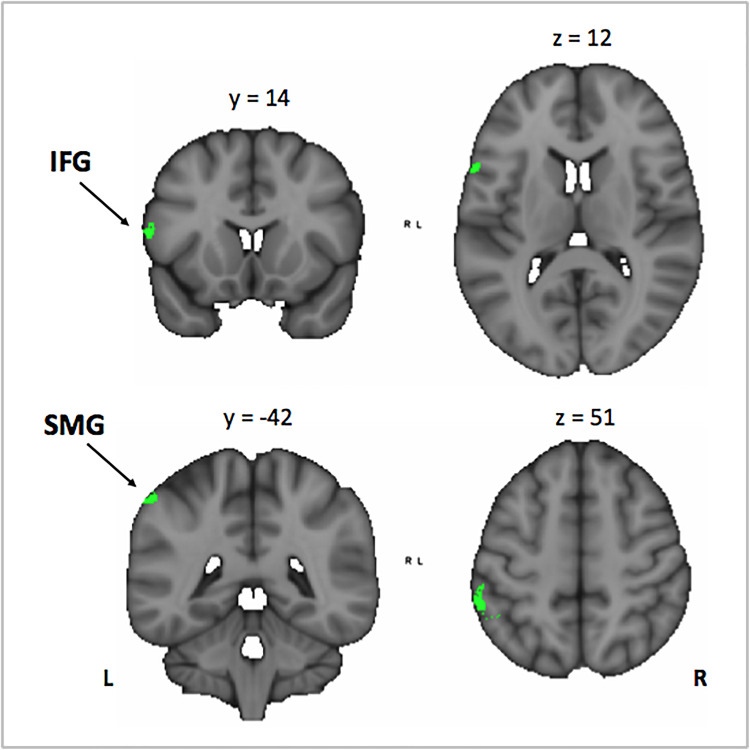
Conjunction analysis between the Mus > Cont contrast of the correlation between the activation in the encoding phase [VSA > P] contrast with the correct responses of each subject in the auditory memory tasks of the VSA condition trials, and the correlation between the years of musical training in the musically trained group in the encoding phase [ASA > P] contrast. Mus, musically trained children; Cont, control children; VSA, visual selective attention; ASA, auditory selective attention; P, passive. IFG, inferior frontal gyrus; SMG, supramarginal gyrus.

In order to investigate the three-term interaction effect found in our behavioral analysis of accuracy, we correlated the activation in the encoding phase of the [VSA > P] contrast with the correct responses of each subject to the AMT in the VSA condition. When comparing the results among groups, we found a higher activation of the left SMG, the SMA, the ACC, the left superior IFG, and the left frontal pole in musically trained children as compared with controls (Figure 5 and Table 5). This effect could be due to a general facilitation for the encoding of auditory stimuli in the musically trained children. If the latter was true, a similar modulation should be found when musically trained children pay attention to auditory stimuli (such as in our ASA condition), and this modulation should correlate with the years of musical training. In order to test this, we made a conjunction analysis between the previous result (Figure 5) and the activity that was found when correlating the years of musical training with the activity during the encoding phase of the [ASA > P] contrast in the musically trained group (Figure 6 and Table 5). We found two points of overlap: the left IFG (specifically the pars opercularis) and the left SMG (Figure 7). To be more certain that these results are specific to auditory processing, we did also explore if there was a correlation between the [ASA > P] contrast and performance on the visual task in the ASA condition, and we did not find any significant activations at our threshold levels (corrected *p* < 0.05).

**TABLE 5:** Peaks of activity of (1) group differences (Mus > Cont children) for the correlation between correct responses on AMT and the contrasts [VSA > P] and (2) musicians for the correlation between years of training and the [ASA > P] contrast.

**Area**	**x**	**y**	**z**	**Z-score**	**Cluster size**	**Corrected *p*-value**
**Mus > Cont for correlation between correct responses on AMT and VSA > P**						
Right supplementary motor cortex	4	−9	55	5.18	5,567	1.19E–06
Left cingulate gyrus, anterior division	0	−9	36	4.92	5,567	1.19E–06
Left supramarginal gyrus	−55	−39	51	5.22	3,228	0.000183
Left frontal pole	−40	57	7	5.35	1,752	0.00849
Inferior frontal gyrus, pars opercularis	−59	10	15	5.1	1,305	0.0327
**Musicians for correlation between years of training and ASA > P**						
Left middle frontal gyrus	−42	10	45	5.06	5,054	3.81E–06
Left inferior frontal gyrus, pars triangularis	−57	26	14	4.65	5,054	3.81E–06
Left middle frontal gyrus	−44	11	45	4.65	5,054	3.81E–06
Left inferior frontal gyrus, pars triangularis/pars opercularis	−52	21	14	4.6	5,054	3.81E–06
Right cingulate gyrus, posterior division	8	−28	37	5	3,216	0.000208
Left precentral gyrus	−15	−32	45	4.91	3,216	0.000208
Left supramarginal gyrus/postcentral gyrus	−53	−30	51	4.63	1,927	0.00556
Right anterior insula	38	13	−7	4.75	1,310	0.0343
Right frontal orbital cortex	35	21	−20	4.5	1,310	0.0343
Right supramarginal gyrus/angular gyrus	44	−47	64	4.98	1,278	0.0379
Right supramarginal gyrus	52	−36	52	4.33	1,278	0.0379

**Mus, musically trained children; Cont, control children; ASA, auditory selective attention condition; VSA, visual selective attention condition; DA, divided attention condition; P, passive condition; AMT, auditory memory task.**

Taken together, our results suggest that musical training facilitates the encoding of auditory stimuli and that this facilitation relies on the left IFG and the left SMG in musically trained children.

## Discussion

Our study investigated the neural dynamics that underlie the improved performance of children who play a musical instrument on a bimodal (auditory/visual) attention and WM task. We found that two mechanisms seem to contribute to this improvement. On the one hand, musically trained children show a higher activation of a more domain-general mechanism, the fronto-parietal control network during encoding phases of all attention conditions. On the other hand, they showed a higher activation of a more domain-specific mechanism of auditory encoding, which includes the left IFG and the left SMG, which are structures that support the phonological loop. These results contribute new knowledge that allows us to better understand how the developing brain is influenced by the achievement of this complex ability. Longitudinal studies on groups paired for general demographics before training suggest that musical training has a “nurture” effect on development and brain plasticity (Schlaug et al., 2005; Kraus et al., 2014; Putkinen et al., 2015; Habibi et al., 2017). Our cross-sectional design does not allow us to address whether there were differences in attention and WM prior to musical training. However, our groups were matched on gender, age, IQ, and socioeconomic status, which allowed us to evaluate the relation between musical training and attention and memory without these confounding factors. Also, our control group was a passive control group that did not engage in another type of activity that also requires self-control, concentration, and regular training. It is though important to mention that longitudinal studies that have included active control groups such as Moreno et al. (2009) and Habibi et al. (2018) have found that musical training has an impact on auditory processing skills and also executive functions such as inhibitory control.

Our behavioral results showed that musically trained children had an overall better performance on both memory retrieval tasks than had control children. These results are in line with other studies that have found that musicians perform better on both AMT and VMT (Rodrigues et al., 2013; Slevc et al., 2016; Talamini et al., 2016). Several studies have proposed that this improvement in visual attention and memory skills is due specifically to music reading and playing in an orchestra (Land and Furneaux, 1997; Rodrigues et al., 2007, 2013). Since all of our musically trained participants read music and played in an ensemble, it is plausible that these specific facets of their musical training may have contributed to the overall better performance of the musically trained children in both memory tasks.

In line with the above-presented behavioral results, our functional brain imaging results showed that musically trained children had higher activation in attentional control related brain areas (e.g., dlPFC and superior PL) (Corbetta and Shulman, 2002) in the encoding phase contrasts of all three active attention conditions (auditory selective [ASA > P], visual selective [VSA > P], and divided attention [DA > P]) than had control children. Musically trained children also showed significantly higher activation of the anterior cingulate cortex (ACC) and the thalamus in the encoding phase contrasts of all active attention conditions than did controls. The ACC is involved with monitoring demands for executive control (Mansouri et al., 2017), and its activity is also associated with the fronto-parietal control network (Power et al., 2011). The thalamus is important for sensory processing and integration (McCormick and Bal, 1994; Cappe et al., 2009), language processing (Crosson, 2013), and memory functions (Kopelman, 2015), and it participates in distributed cognitive control (Halassa and Kastner, 2017). A recent study also showed that the functional connectivity of the thalamocortical network is reorganized in musicians (Tanaka and Kirino, 2017). This latter study showed that auditory areas are more strongly connected with the left thalamus in musicians as compared with controls. Our results also expand on the results obtained by Pallesen et al. (2010) with young adult musicians, who showed that the cognitive control network was enhanced during auditory WM in musicians. Taken together, our results suggest that playing a musical instrument boosts cognitive control networks such as the fronto-parietal attention network and the thalamus during the encoding phase and that this subserves the improved memory capacity for auditory and visual stimuli shown by musically trained children in our task.

Another of our behavioral results for accuracy showed a three-way interaction between the factors group, attention condition, and memory task. This interaction was given by the group differences in the correct responses to the AMT after the VSA, with the musically trained children showing significantly better performance than control children. This result shows that even though participants were instructed to pay attention only to the visual stimuli in this condition, musically trained children were still able to encode and remember the auditory stimuli that were presented during the encoding phase far better than control children. When we correlated the brain activity in the encoding phase of VSA (contrast [VSA > P]) with the correct responses on the AMT for this attention condition, we found significant differences among groups, with musicians showing higher activation in the left SMG, among others.

We hypothesized that this behavioral effect could be due to a facilitated encoding of auditory stimuli in the musically trained group. We reasoned that if this was true, a similar modulation should be found when musically trained children pay attention to auditory stimuli (such as in our ASA) and that this modulation should correlate with the years of musical training. In order to test this, we checked if there was an overlap between the previous result and the activity that correlated with the years of musical training in the musically trained group during the encoding phase of the ASA condition ([ASA > P] contrast). We found two points of overlap: the left IFG (specifically the pars opercularis) and the left SMG.

Both these areas are multimodal association areas known to support the phonological loop. The phonological loop is part of the WM system involved in auditory processing; in particular, it is thought to be implicated in establishing auditory–motor connections (Baddeley, 2003; Schulze and Koelsch, 2012). Importantly, it has been shown that these areas are core structures involved in both tonal and verbal auditory WM (Koelsch et al., 2009). In fact, it has been observed that these areas are more activated in musicians than non-musicians during tonal WM tasks (Schulze et al., 2011).

In general, the IFG, specifically Broca’s area, is known to be part of the language network and is involved in the perception and vocalizations of speech (Aboitiz, 2017). Notably, this area is also important for the recognition of musical auditory patterns (Chiang et al., 2018). It has also been suggested that the IFG contributes to memory formation (Tang et al., 2018). On the other hand, it has been shown that musicianship seems to have an impact on the structure of the left IFG. Abdul-Kareem et al. (2011) found that increased gray matter volume of left pars opercularis in male orchestral musicians correlated positively with years of musical performance. One could speculate then that our functional finding that subserved auditory memory encoding in our musically trained group could eventually lead to increased gray matter in this area.

On the other hand, the left SMG has been shown to participate in pitch memory (Gaab et al., 2003; Ellis et al., 2013). Notably, research using transcranial direct current stimulation (tDCS) (Vines et al., 2006; Schaal et al., 2017) and transcranial magnetic stimulation (TMS) (Schaal et al., 2015) have implied that the left SMG is causally involved with pitch memory processing. Another recent study that included cross-sectional and longitudinal data also showed that the left SMG is involved in music processing in musically trained children and adults (Ellis et al., 2013). Participants in Ellis et al. (2013) solved the same/different melodic and rhythmic discrimination task. Similar as in our results, they found that activation in the left SMG was related to cumulative hours of musical practice in both tasks for children and adults. Our results suggest that musical training facilitated the encoding of auditory stimuli in the musically trained children and that this facilitation relied on the left IFG and the left SMG. This results could also help to interpret the positive impact that musical interventions have on children with dyslexia (Habib et al., 2016), and this and the abovementioned results also support the overlap and attention conditions of the OPERA hypothesis proposed by Patel for the benefit of musical training on the neural encoding of speech (Patel, 2011).

Interestingly, the IFG and SMG, which we found to be involved specifically in auditory encoding in our musically trained group, have also been shown to be involved in visual stimuli processing. For example, Broca’s area in the IFG has been shown to be activated to a greater extent by visually presented sentences when compared with spoken sentences (Carpentier et al., 2001), and the SMG has been causally involved in visual word recognition (Stoeckel et al., 2009). These results suggest that it is plausible that the increased functioning of these areas in musicians could eventually impact their visual processing. Effectively, it has also been found that Broca’s area supports enhanced visuospatial cognition in professional orchestral musicians (Sluming et al., 2007).

Our results do not support the neural efficiency hypothesis, which states that subjects with better performance show lower brain activation than individuals with lower performance when working on the same cognitive tasks (Dunst et al., 2014). Evidence has suggested that this phenomenon also seems to be a function of the amount and quality of learning; this means that the specialization of functioning is reached over time (Neubauer and Fink, 2009). It is probable that in the case of this study, the children are still in the “training phase” of the functions, and that is why we see a higher functioning of the networks. This would have to be tested in other experiments.

Taken together, our results describing the neural dynamics the underlie the improved performance of musically trained children in our attention task suggest that musical training improves the allocation of attentional resources by increasing the functioning of the fronto-parietal control network and facilitating the encoding of auditory stimuli. This latter benefit is due to the years of training and depends on the function of left IFG and left SMG, structures that also support the phonological loop. Our results could be relevant for educational policies, and they also suggest that musical training could be used as a non-pharmacological intervention strategy for children with attentional problems in order to improve their overall functioning in daily life.

## Data Availability Statement

The raw data supporting the conclusions of this article will be made available by the authors, without undue reservation, to any qualified researcher.

## Ethics Statement

The studies involving human participants were reviewed and approved by the Ethics Committee of the Medical Faculty of the Pontificia Universidad Católica de Chile. Written informed consent to participate in this study was provided by the participants’ legal guardian/next of kin.

## Author Contributions

LK, FZ, MES, and FA designed the experiment. LK and MES created the stimuli. LK and PB programmed the experiment. LK and JL-V recruited the subjects. JL-V and XS evaluated the subjects. LK and FZ conducted the experiments. LK analyzed the data. LK, FZ, PB, GS, and FA discussed the results. All authors provided revisions and critical feedback on the final draft of the manuscript.

## Conflict of Interest

The authors declare that the research was conducted in the absence of any commercial or financial relationships that could be construed as a potential conflict of interest.

## Footnotes

1 http://www.mrivideo.com/

2 http://www.mrivideo.com/

3 https://fsl.fmrib.ox.ac.uk/fsl
